# Exploring the Psychometric Properties of the Farsi Version of Quality of Life Kindl Questionnaire for 4-7 Year-Old Children in Iran

**Published:** 2016

**Authors:** Maryam ROJHANI SHIRAZI, Seyed Hassan TONEKABONI, Eznollah AZARGASHB, Mehdi DERAKHSHANNIA, Elham AGHDASTA

**Affiliations:** 1 Clinical Research Development Center, Mofid Children Hospital, Shahid Beheshti Medical University, Tehran, Iran; 2 Department of Community Medicine, Faculty of Medicine, Shahid Beheshti University of Medical Sciences, Tehran, Iran; 3 Master of Counseling, Allameh Tabatabai University, Tehran, Iran

**Keywords:** Psychometric properties, Validity, Reliability, Quality of life, Kindl Questionnaire, Children

## Abstract

**Objective:**

The aim of this study was to translate and validate the psychometric properties of the Quality of Life Kindl questionnaire.

**Materials & Methods:**

Parents of 4-7 yr-old healthy and ill children referred to Mofid Children Hospital in Tehran in 2013, Iran were sampled randomly in two groups each of which 130 people. After translation, the questionnaie’s validity and reliability was evaluated and was confirmed for face and content validity. Questionnaire was also completed by two (one healthy and one ill) groups for which inclusion criteria included consent of the parents, age of the children being beween 4 and 7 yr, and presence of the child in a nursery school, kindergarten, school or any class at least for one month. Exclusion criteria were inability of the parents in answering the questions accurately. Inclusion criterion for the ill group was having chronic cardiac, neurologic, hematologic, or respiratory diseases, lasting longer than 3 months for which they were followed up in outpatient clinic in the hospital. The reliability of questionnaire was measured by the Cronbach’s alpha.

Data were analyzed using factor analysis, Spearman’s correlation coefficient, Mann-Whitney and Chi-square test.

**Results:**

The reliability was 0.85 and 0.81 in healthy and ill groups, respectively. The results of factor analysis showed that each of eight subscales of questionnaire had acceptable construct validity. Only two of 52 questions of the questionnaire did not have proper correlation coefficient.

**Conclusion:**

Quality of Life Kindl Questionnaire is a valid and reliable test for assessing healthy and ill children in Iran.

## Introduction

The World Health Organization (1) has considered the quality of life (QoL) as a useful supplementary concept to the traditional health and functioning concepts.

Therefore, it is introduced as a necessary adjunct to “include a measure of the person’s physical health, a measure of physical, social and psychological functioning, and a measure of quality of life”. Health related quality of life (HRQoL) in turn, has been defined as dimensions of general quality of life that can affect physical or mental health (2). Self-assessed health status can be a more powerful predictor of mortality and morbidity than many objective measures of health (3). 

Most of the work done on assessing quality of life has concentrated on the adult population and just a few studies have considered younger population (4-6). To measure the quality of life of the 4-7 year-old children, different tools have been designed in different countries, which in addition to the problems in determining their reliability and validity; they are not consistent with Iran’s culture. On the other hand, a proper tool, which is consistent with Iran’s culture, is not designed yet. 

Whereas based on WHO definition, cultural context can influence HRQoL. Therefore, there is a need for a valid and reliable tool that is congruent with Iran’s society. 

General quality of life Kindl questionnaire is designed by Ravens-Sieberer and Bullinger (6, 7) in Germany to investigate the quality of life related to the health in children. This questionnaire is applied to evaluate eight different areas of life (physical health, emotional health, self-esteem, family, social relationships, school, some important questions and hospitalization of the child in the present time or his/her chronic disease). Kindl is a short, flexible and methodologically suitable instrument, which is translated into more than 27 languages and has been employed in numerous international studies, yielding sound psychometric properties.

So far some efforts has been done to investigate the consistency of the concepts among Western and Eastern cultures and whether this questionnaire remains valid after being translated into other languages (8, 9) and a few instruments have been able to prove valid after being translated into the target language. However, so far no study has translated and evaluated Quality of Life Kindl questionnaire into Farsi.

This study was implemented to translate and validate quality of life Kindl questionnaire for 4-7 yr-old children that is completed by parents. Having an appropriate tool to measure the quality of life can help the researchers in medical sciences evaluate the consequences and the efficacy of therapeutic and health care measures. This study can also be used as a foundation for validating and developing other tools for research data gathering. 

Hence, current research aims at determining validity and reliability of the Farsi version of the quality of life Kindl questionnaire for 4-7 year-old children.

## Materials & Methods

**Instrument**

General quality of life Kindl questionnaire was developed by Bullinger et al. (10) and revised by Sieberer and Bullinger (6) in Germany to investigate the quality of life related to the health in children. This questionnaire is applied to evaluate 8 different areas of life: physical health (4 items), emotional health (4 items), self-esteem, family (4 items), social relationships (4 items), school (4 items), some important questions (22 items) and hospitalization of the child in the present time or his/her chronic disease (6 items). It was developed as a short, flexible, and methodologically suitable instrument, which can be completed by both the child and the parent. 

It is available for a wide range of the healthy or ill children and adolescents. The questionnaire is implemented on 3000 healthy and chronically ill children and their parents and has been applied in numerous studies yielding a high degree of reliability (Cronbach’s.70 for most of the sub-scales and samples) and a satisfactory convergent validity of the procedure, beyond which the acceptance of the measure by children and adolescents is high (6). It is translated into more than 27 languages. 

In current study, Farsi version of the quality of life Kindl questionnaire for 4-7 year-old children was employed.

**Participants**

Parents of the 4-7 yr-old children who referred to Tehran’s Mofid Pediatric Hospital were the studied population from which both the healthy and ill group were selected in 2013. Overall, 130 parents were selected from healthy children group and 130 from ill ones. For sampling, we used random sampling method. Using the formulan=Z1-α/2+Z1-β12 Ln 1+r1-r2+3 and considering α = 0.05 and β = 0.20 and r = 0.80, and number of items in the questionnaire (52 items), the sample size was calculated to be 260 people in which the proportion of the healthy and ill subjects was equal. 

Parents completed a consent letter to participate in the study.

**Data analysis method**

To investigate the validity of the questionnaire in internal consistency aspect, Chronbach’s alpha was used. In addition, factor analysis was employed to investigate construct validity. To compare quality of life in two groups of healthy and ill groups, Mann-Whitney and X2 tests, and to measure the correlation among the questions and the areas of the questionnaire, Spearman’s correlation coefficient was used.

**Implementation method**

This study was implemented in two phases: 1. Translation phase; 2. Implementation phase. In the first stage, the questionnaire was translated from the original language for which following 7 stages were covered: 

1. Translation of the questionnaire from English into Farsi: Questionnaire was translated from English into Farsi by two proficient translators who had excellent command of both Farsi and English (forward translation).

2. Combining and integrating the preliminary translations into a single translation: In this stage, translated versions were reviewed by other translators who have not participated former stages. 

The differences and conflicts were corrected and at last, the final translated version was prepared by integrating the preliminary translations.

3. Translation of the final translated version from Farsi into English: The final version whose translation was completed in the last stage was translated into Farsi by a translator who had an excellent command of both Farsi and English and had not participated in other stages of the translation (backward translation) 

4. Revising translated version from Farsi into English: In this stage, the final questionnaire, which was translated from Farsi into English, was revised. This was done by a translator (familiar with the concepts and expert in translating the questionnaires) who mastered both Farsi ad English. This version was compared with the original version to revise the dissonances if observed any, but since the dissonances were negligible, the corrections and 

5. Modifying and editing: In this stage, experts reviewed the translated text.

6. Determining validity and reliability of the translated questionnaire: In current study, Kindl questionnaire’s validity was determined by content validity method. The questionnaire was presented to 10 referees and they judged it based on the relatedness, clearness and simpleness. In the next stage, based on the experts’ ideas, interview question were reviewed and modified. This way, face validity and content validity were confirmed and determined. In this research, Chronbach’s alpha was used to determine the reliability of the quality of life questionnaire for 4-7 yr-old children. To investigate the construct validity of the questionnaire, factor analysis was employed.

7. Presenting the final questionnaire: In this stage, all the translation process and its cultural adjustment were prepared along with the final version. 

To select the sample, WHO guideline was used to determine the psychometrics of the quality of life questionnaire. In this guideline, it is recommended that half of the participants should be female and the other half should be male.

**Inclusion criteria: **Getting a consent letter from the parents of 4-7 year-old children and having a child who has attended nursery school, kindergarten, school or any other kind of class in which child has had a responsibility for at least one month.

**Exclusion criteria: **Disability of the parents of 4-7 yearold children in answering the questions accurately due to mental retardation or deafness, muteness etc. and mental disability in answering questions correctly or having a child with deafness, or mentally retarded in mild, moderate or severe levels.

**Operational definition of the groups: **Healthy child: A child who did not have a specific disorder. Ill child: A child who was suffering from a chronic disease (more than three months) or was hospitalized following a chronic disease when parents were filling the questionnaire.

In the next stage, the questionnaire was completed for two groups of people. When filling the questionnaire, researcher, after introducing herself and explaining the goals of the study and reassuring the parents about the confidentiality of the data, collected the information. In addition to inquiry to make the questions understood and to simplify some questions for mothers, in some cases complementary information was also provided. In the next stage, questionnaire’s data was entered to the SPSS software. In statistical analysis, in scoring of the questions, to calculate the total score of the questionnaire, some questions’ score (indicated in the following part) was inverted (negative weighing).

Negative weighed questions included 1, 2, 3, 6, 7, 8, 15, 16, 20, 24, 25, 28, 31, 34, 36, 38, 39, 41, 44, 45, 46, 47, 48, 50, 51, and 52.

## Results

Content validity of the questionnaire was confirmed by the correlation coefficients of 0.89, 0.78 and 0.76 based on relatedness, clearness and simpleness indexes. 

A pilot study was also implemented on a sample of 30 people. This questionnaire was completed by 30 parents of the 4-7 yr-old children (15 healthy and 15 ill) for which Chronbach’s alpha was calculated to be 0.78 which is higher than 0.7 and therefore, acceptable. 

From 260 people of the sample, 130 were healthy and 130 were ill whose demographic specifications are presented in the Tables 1-3. 

Table 1 shows that in the healthy group, number of the girls was slightly higher than boys were and in the ill group, number of the boys was higher. In total, number of the boys was higher than the number of the girls. 

Besides, in healthy and ill groups, gender distribution was not significantly different (P = 0.106).

Table 2 shows that both in healthy and ill groups, 4 year old children had the highest and 7 yr-old children had the lowest frequency of the participants of the study, and healthy and ill groups were not significantly different according to age (P = 0.201).

Table 3 shows that in every group of healthy and ill subjects, the highest frequency in parents’ education relates to high school and the lowest frequency relates to elementary school and the two groups didn’t have a significant difference in education (P = 0.455). 

Area means in healthy and ill groups have been compared which except for emotional area and total score of the questionnaire, there is no significant difference in other areas (Table 4).

To investigate the construct validity, factor analysis was employed which is presented in Table 5. Results of the analysis showed that 8 areas (factors) of the questionnaire have factor weights of higher than 1 and this indicates that questionnaire has acceptable construct validity. Percentage of rotated and accumulated variance of every area is presented in the Table 5.

To investigate the reliability of the questionnaire in internal consistency, Chronbach’s alpha has been used which is presented in the Table 6. Amount of Chronbach’s alpha in total the questionnaire (including illness) was 0.81, which is higher than 0.7. This index also was 0.7 in sixth area (school) and 0.74 in area 7 (some important questions). But in areas 1 (physical health), 2 (emotional health), 3 (self esteem), 4 (family), 5 (social relationships) and 8 (illness) it was calculated to be 0.58, 0.38,0.47, 0.58, 0.34 and 0.52 respectively which were lower than 0.7, which might be the result of low number of questions.

Every area’s Chronbach’s alpha along with total score in the healthy and ill groups is presented. Total Chronbach’s alpha is 0.85 and 0.81 in healthy and ill group both of which are above 0.7 (Table 7).

To investigate correlation of the questions and areas with the total score of the questionnaire, Spearman’s correlation coefficient was used (Table 8) and shows that correlation coefficient in each of 8 areas in healthy, ill and total groups was satisfactory. Besides, through the all the subjects studied, 50 out of 52 questions had satisfactory correlation coefficient and just two questions – question 20 (question 4 in social relationships area: My child felt to be different from the others) and question 51 (question 5 in illness area: My child didn’t let the others find out about her illness) – had a low correlation coefficient from the total score of the questionnaire, so they need to be revised.

## Discussion

The current study tried to evaluate the psychometric properties of quality of life Kindl questionnaire for 4-7 yr-old children in Farsi language and adapt it with cultural conditions of Iran. According to Table 7, total Chronbach’s alpha was 0.85 for healthy and 0.81 for ill group both of which were satisfactory and acceptable. Furthermore, this index in area 6 (school) and 7 (some important questions) was more than 0.7 so, it was acceptable (Table 6). However, in other six areas (physical health, emotional health, self-esteem, family, social relationships and illness) it was less than 0.7, which might be a result of having too few questions in those areas, or high sensitivity of the questions. In a study conducted to standardize WHO Questionnaire for Quality of Life (WHOQOL BREF), among the four areas evaluated, three – physical health, mental health and environmental health – had Chronbach’s alpha of over 0.7, but about the social relationships area, this index was 0.55 which is consistent with our results (11). 

The range of Chronbach’s alpha in five factors was 0.81 to 0.92 in another study (12). In addition, for designing and measuring quality of life in 6-16 year-old children suffering from allergies in England, three dimensions (areas) were investigated (13). Chronbach’s alpha in behavioral problems, disease symptoms, and emotional problems was calculated to be 0.88, 0.92 and 0.73, respectively, and total Chronbach’s alpha was 0.95 (13). 

As it can be seen in these three studies, they have a higher Chronbach alpha than current study, and this is probably due to the higher age of the children under study and having the same kind of illness. Factor analysis results showed that all the eight areas (factors) of the questionnaire were confirmed in assessing the quality of life for 4-7 year-old children, which indicates that the questionnaire has acceptable construct validity. It can be considered as a valid questionnaire to be used for 4-7 yr old Iranian children.

In addition, correlation coefficient in all eight groups of healthy, ill subjects and total was satisfactory. Besides, 50 out of 52 questions of the questionnaire had acceptable correlation coefficient, so structure of the questionnaire seems to be acceptable. Only two questions – question 20 (question 5 in social relationships area: My child felt to be different from the others) and question 51 (question 5 in illness area: My child did not let the others find out about her illness) – had a low correlation coefficient from the total score of the questionnaire, which might be due to incomprehensibility of these two questions for 4-7 age range of the children and it is recommended that these two questions are omitted or modifies. 

Availability of the children with chronic diseases and children in nursery age was one of the limitations of this study, which imposed spending far more time to implement the study. Besides, due to the large number of the data, entering them in the SPSS software and applying analyses was a burdensome task. 

**In conclusion, **translated version of the quality of life Kindl questionnaire has acceptable validity and reliability. On the other hand, this tool has been translated into 18 different languages in different parts of the world, and so, it is possible to compare the results of the projects in different countries. Since it has satisfactory validity, it can be applied with healthy and ill children.

**Recommendations**

After consulting with the experts, the authors have been convinced that modifying two questions – questions 20 and 51 – and making them more comprehensible is inevitable. It is also recommended that extensive studies be implemented in on the children of older ages, on larger number of ills and in different centers.

**Table 1:** Gender Frequency Distribution of the Studied Subjects in Healthy and Patient Groups

**Groups**	**Girls**	**Boys**	**Total**
**Healthy**	68 (52.3%)	62 (47.7%)	130 (100%)
**Patient**	54 (41.5%)	76 (58.5%)	130 (100%)
**Total**	122 (46.9%)	138 (53.1%)	260 (100%)

**Table 2:** Age Distribution of the People Studies in Healthy and Unhealthy Groups

**Groups**	**Age (years)**				**Total**
	**4**	**5**	**6**	**7**	
**Healthy**	54 (41.5%)	37 (28.5%)	22 (16.9%)	17 (13.1%)	130 (100%)
**Patient**	41 (31.5%)	34 (26.2%)	31 (23.8%)	24 (18.5%)	130 (100%)
**Total**	95 (36.5%)	71 (27.3%)	53 (20.4%)	41 (15.8%)	260 (100%)

**Table 3:** Education Distribution of The Parents in Healthy and Patient Groups

**Groups**	**Parents’ education**				**Total**
	**Elementary school**	**Guidance school**	**High school**	**University**	
**Healthy**	8 (6.2%)	17 (12.4%)	72 (55.8%)	33 (25.6%)	130 (100%)
**Patient**	10 (7.7%)	22 (16.9%)	74 (56.9%)	24 (18.5%)	130 (100%)
**Total**	18 (6.9%)	39 (14.7%)	146 (56.4%)	57 (22%)	260 (100%)

**Table 4:** Comparison of Mean Values and Standaerd Deviation of Different Areas of Kindl Questionnaire in Healthy and Patient Groups (Except Illness Area

**Groups**		**Number**	**Mean**	**Standard deviation**	**P-Value**
**Physical health**	**Healthy**	130	3.95	0.61	0.068
	**Patient**	130	3.81	0.61	
**Emotional health**	**Healthy**	130	3.62	0.62	0.003
	**Patient**	130	3.39	0.62	
**Self esteem**	**Healthy**	130	3.52	0.54	0.497
	**Patient**	130	3.46	0.50	
**Family**	**Healthy**	130	3.26	0.60	0.497
	**Patient**	130	3.21	0.63	
**Social relationships**	**Healthy**	130	3.85	0.55	0.415
	**Patient**	130	3.79	0.51	
**School**	**Healthy**	130	4.12	0.58	0.166
	**Patient**	130	4.00	0.71	
**Some important questions**	**Healthy**	130	3.69	0.35	0.072
	**Patient**	130	3.62	0.33	
**Total score of the questionnaire**	**Healthy**	130	3.71	0.31	0.034
	**Patient**	130	3.63	0.29	

**Table 5:** Factor Analysis Results of Kindl Questionnaire

**Areas**	**Sum of squares of rotated factor weights**	**Rotated variance percentage**	**Accumulated variance**
**Physical health**	6.26	13.61	13.61
**Emotional health**	3.26	7.10	20.71
**Self esteem**	2.93	6.37	27.09
**Family**	2.18	4.74	31.83
**Social relationships**	2.04	4.43	36.27
**School**	1.69	3.67	39.94
**Some important questions**	1.51	3.28	43.23
**Illness**	1.41	3.08	46.31

**Table 6:** Reliability Coefficient of Whole the Kindl Questionnaire and Its Different Areas (Chronbach’s Alpha

**Areas**	**Chronbach’s alpha**
**Physical health**	0.58
**Emotional health**	0.38
**Self esteem**	0.47
**Family**	0.58
**Social relationships**	0.34
**School**	0.70
**Some important questions**	0.74
**Illness**	0.52
**Whole the questionnaire (including the illness)**	0.81
**Whole the questionnaire (excluding the illness)**	0.83

**Table 7:** Reliability Coefficients of Kindl Questionnaire in Healthy and Unhealthy Groups in Different Areas

**Areas**	**Healthy Group**	**Patient Group**
**Physical health**	0.67	0.49
**Emotional health**	0.38	0.35
**Self esteem**	0.55	0.38
**Family**	0.61	0.57
**Social relationships**	0.37	0.28
**School**	0.72	0.69
**Some important questions**	0.76	0.72
**Illness**	-	0.52
**Total**	0.85	0.81

**Table 8:** Correlation Coefficient of The Questions and Different Fields of The Questionnaire in Healthy Group, Patient Group and Total

**Items**	**Healthy group**		**Patient group**		**Total**	
	**Correlation coefficient**	**P-value**	**Correlation coefficient**	**P-value**	**Correlation coefficient**	**P-value**
**1**	0.317	<0.001	0.195	0.027	0.263	<0.001
**2**	0.328	<0.001	0.182	0.038	0.251	<0.001
**3**	0.274	0.002	0.409	<0.001	0.347	<0.001
**4**	0.384	<0.001	0.370	<0.001	0.383	<0.001
**5**	0.504	<0.001	0.491	<0.001	0.503	<0.001
**6**	0.473	<0.001	0.513	<0.001	0.500	<0.001
**7**	0.376	<0.001	0.336	<0.001	0.370	<0.001
**8**	0.360	<0.001	0.260	0.003	0.315	<0.001
**9**	0.365	<0.001	0.200	0.023	0.276	<0.001
**10**	0.359	<0.001	0.260	0.003	0.314	<0.001
**11**	0.372	<0.001	0.313	<0.001	0.354	<0.001
**12**	0.209	0.017	0.160	0.068	0.187	0.003
**13**	0.494	<0.001	0.408	<0.001	0.454	<0.001
**14**	0.403	<0.001	0.165	0.060	0.272	<0.001
**15**	0.434	<0.001	0.426	<0.001	0.421	<0.001
**16**	0.414	<0.001	0.359	<0.001	0.400	<0.001
**17**	0.205	0.019	0.327	<0.001	0.264	<0.001
**18**	0.384	<0.001	0.187	0.033	0.300	<0.001
**19**	0.341	<0.001	0.241	0.006	0.300	<0.001
**20**	0.118	0.181	0.035	0.692	0.095	0.127
**21**	0.257	0.003	0.353	<0.001	0.304	<0.001
**22**	0.197	0.025	0.329	<0.001	0.277	<0.001
**23**	0.203	0.021	0.314	<0.001	0.272	<0.001
**24**	0.399	<0.001	0.265	0.002	0.328	<0.001
**25**	0.524	<0.001	0.625	<0.001	0.574	<0.001
**26**	0.482	<0.001	0.216	0.013	0.354	<0.001
**27**	0.332	<0.001	0.187	0.033	0.267	<0.001
**28**	0.473	<0.001	0.427	<0.001	0.459	<0.001
**29**	0.284	0.001	0.273	0.002	0.279	<0.001
**30**	0.266	0.002	0.152	0.084	0.214	0.001
**31**	0.281	0.001	0.410	<0.001	0.335	<0.001
**32**	0.579	<0.001	0.619	<0.001	0.601	<0.001
**33**	0.374	<0.001	0.265	0.002	0.324	<0.001
**34**	0.333	<0.001	0.279	0.001	0.301	<0.001
**35**	0.243	0.005	0.215	0.014	0.232	<0.001
**36**	0.456	<0.001	0.391	<0.001	0.420	<0.001
**37**	0.437	<0.001	0.259	0.003	0.341	<0.001
**38**	0.064	0.469	0.253	0.004	0.171	0.006
**39**	0.580	<0.001	0.542	<0.001	0.570	<0.001
**40**	0.525	<0.001	0.440	<0.001	0.500	<0.001
**41**	0.121	0.169	0.261	0.003	0.171	0.006
**42**	0.345	<0.001	0.261	0.003	0.309	<0.001
**43**	0.406	<0.001	0.269	0.002	0.338	<0.001
**44**	0.400	<0.001	0.398	<0.001	0.411	<0.001
**45**	0.359	<0.001	0.362	<0.001	0.355	<0.001
**46**	0.455	<0.001	0.470	<0.001	0.465	<0.001
**47**	-	-	0.368	<0.001	0.368	<0.001
**48**	-	-	0.320	<0.001	0.320	<0.001
**49**	-	-	0.261	0.003	0.261	0.003
**50**	-	-	0.313	<0.001	0.313	<0.001
**51**	-	-	0.104	0.140	0.104	0.140
**52**	-	-	0.225	0.010	0.225	0.010
**Physical health**	0.454	<0.001	0.451	<0.001	0.460	<0.001
**Emotional health**	0.690	<0.001	0.644	<0.001	0.674	<0.001
**Self esteem**	0.486	<0.001	0.400	<0.001	0.446	<0.001
**Family**	0.639	<0.001	0.522	<0.001	0.580	<0.001
**Social relationships**	0.423	<0.001	0.325	<0.001	0.380	<0.001
**School**	0.350	<0.001	0.434	<0.001	0.400	<0.001
**Some important questions**	0.930	<0.001	0.900	<0.001	0.915	<0.001
**Disease**	-	-	0.482	<0.001	0.482	<0.001
